# Interactions with Iridophores and the Tissue Environment Required for Patterning Melanophores and Xanthophores during Zebrafish Adult Pigment Stripe Formation

**DOI:** 10.1371/journal.pgen.1003561

**Published:** 2013-05-30

**Authors:** Larissa B. Patterson, David M. Parichy

**Affiliations:** Department of Biology, University of Washington, Seattle, Washington, United States of America; Stanford University School of Medicine, United States of America

## Abstract

Skin pigment patterns of vertebrates are a classic system for understanding fundamental mechanisms of morphogenesis, differentiation, and pattern formation, and recent studies of zebrafish have started to elucidate the cellular interactions and molecular mechanisms underlying these processes. In this species, horizontal dark stripes of melanophores alternate with light interstripes of yellow or orange xanthophores and iridescent iridophores. We showed previously that the highly conserved zinc finger protein Basonuclin-2 (Bnc2) is required in the environment in which pigment cells reside to promote the development and maintenance of all three classes of pigment cells; *bnc2* mutants lack body stripes and interstripes. Previous studies also revealed that interactions between melanophores and xanthophores are necessary for organizing stripes and interstripes. Here we show that *bnc2* promotes melanophore and xanthophore development by regulating expression of the growth factors Kit ligand a (Kitlga) and Colony stimulating factor-1 (Csf1), respectively. Yet, we found that rescue of melanophores and xanthophores was insufficient for the recovery of stripes in the *bnc2* mutant. We therefore asked whether *bnc2*-dependent iridophores might contribute to stripe and interstripe patterning as well. We found that iridophores themselves express Csf1, and by ablating iridophores in wild-type and mutant backgrounds, we showed that iridophores contribute to organizing both melanophores and xanthophores during the development of stripes and interstripes. Our results reveal an important role for the cellular environment in promoting adult pigment pattern formation and identify new components of a pigment-cell autonomous pattern-generating system likely to have broad implications for understanding how pigment patterns develop and evolve.

## Author Summary

Pigment patterns are some of the most distinctive, diverse and aesthetically pleasing traits of vertebrates. In turn, these patterns offer an outstanding opportunity to understand the mechanisms underlying the development of adult form and how such mechanisms change evolutionarily. Among the especially wide-ranging pigment patterns of teleost fishes, the most thoroughly studied example is the horizontal striping of zebrafish. In this species, stripes result from the precise arrangements of three classes of pigment cells: black melanophores, yellow or orange xanthophores and silvery iridophores. Previous studies showed that stripe formation requires interactions between melanophores and xanthophores. Nevertheless, roles for factors in the tissue environment experienced by pigment cells, as well as roles for iridophores in the pattern-forming process, have remained largely unexplored. Here we identify molecular mechanisms through which pigment cells are supported as the pattern develops. We further show that stripe development requires not only interactions between melanophores and xanthophores but iridophores as well, identifying a complex, pattern-generating system that may be applicable to understanding patterns and diversity across species. Our findings thus highlight the critical role of the “canvas” on which the pattern is painted, as well as the developmental artistry through which the “paints” are applied.

## Introduction

The pigment patterns of teleost fishes are extraordinarily diverse and have important functions in mate choice, shoaling and predation avoidance [1]–[4]. These patterns result from the spatial arrangements of several classes of pigment cells including black melanophores that contain melanin, yellow or orange xanthophores with pteridines and carotenoids, and iridescent iridophores having purine-rich reflecting platelets [5]–[7]. In recent years, mechanisms underlying pigment pattern development, as well as pattern diversification among species, have started to be elucidated. Much of this work has used the zebrafish *Danio rerio* or its relatives [5], [8].

In zebrafish, two distinct patterns develop over the life cycle. The first of these arises in embryos and persists through early larval stages [9]–[14]. Pigment cells of this early larval pattern develop directly from neural crest cells and generate stripes of melanophores at the edges of the myotomes and at the horizontal myoseptum; a few iridophores occur within these stripes whereas xanthophores are scattered widely over the body. The second, adult pigment pattern begins to develop during the larval-to-adult transformation and largely replaces the early larval pigment pattern [15]. Most cells comprising the adult pigment pattern differentiate from post-embryonic latent precursors, with the best studied of these cells, the melanophores, differentiating primarily between ∼2–4 weeks post-fertilization [16]–[19]. By the end of this period a juvenile pigment pattern has developed consisting of two dark stripes of melanophores bordering a light interstripe of xanthophores and iridophores. As the fish grows, stripes and interstripes are added dorsally and ventrally. In the adult, some iridophores are also found within the melanophore stripes, including an ultrastructurally distinct class of these cells having large, rather than small, reflecting platelets [20]. Cells comprising the body stripes and interstripes are found within the hypodermis [20], [21], between the epidermis and the myotome; pigment cells are also found in the scales, fins, and epidermis.

Previous studies showed that development of adult stripes and interstripes requires interactions between different pigment cell classes. For example, *colony stimulating factor 1 receptor* (*csf1r*) encodes a receptor tyrosine kinase required for xanthophore survival and migration [22]; *csf1r* mutants are deficient in xanthophores and also have disorganized melanophores. Yet stripes and interstripes could be restored in these fish by reintroducing xanthophores, either through cell transplantation or in the context of temperature-shift experiments using a temperature-sensitive *csf1r* allele [23], [24]. These experiments suggested that xanthophores are required to organize melanophores into stripes. Subsequent studies identified additional short-range and long-range interactions between these cell types [25]–[27], the dynamics of which are consistent with a process of local self-activation and lateral inhibition, sometimes referred to as a “Turing mechanism” [28]–[30]. Such models often assume single, diffusible activators and inhibitors, though other cellular mechanisms can be accommodated as well. Indeed, theoretical and empirical analyses of melanophore and xanthophore behavior can recapitulate a wide range of pattern variants [31], [32].

**Figure 1:**
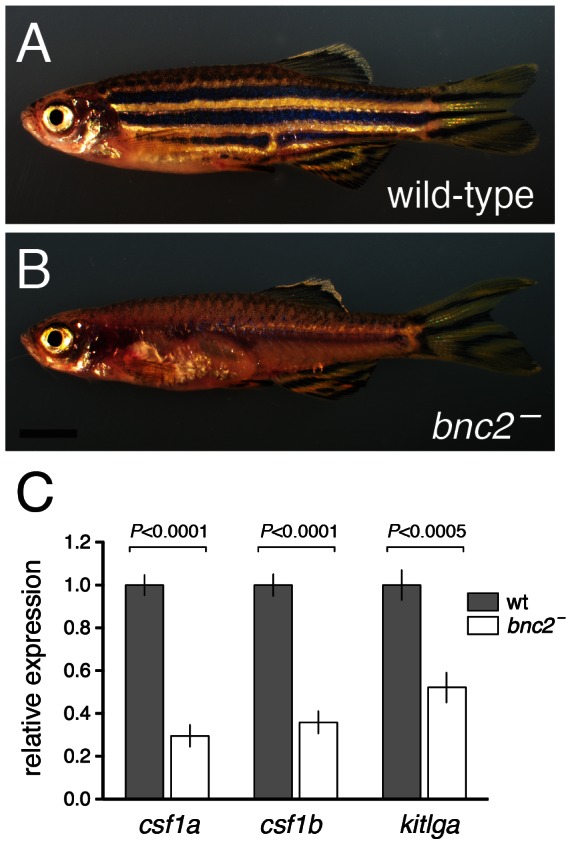
*bnc2* mutants exhibited reduced expression of melanogenic and xanthogenic factors. (A) Wild-type. (B) Homozygous *bnc2* mutant. (C) Quantitative RT-PCR for *csf1a*, *csf1b*, and *kitlga* revealed significantly reduced transcript abundances in skins isolated from 8.5 SSL *bnc2* mutants as compared to stage-matched, wild-type *bnc2*/+ siblings. Shown are means±SE. Values are derived from 3 replicate experiments each consisting of 3 biological replicates for each genotype (*n* = 9 larvae total per genotype). Scale bar: in (B) 3 mm for (A,B).

Despite the importance of interactions among pigment cells, the environment in which these cells reside also influences their development and patterning. Such effects are illustrated dramatically by mutants for *basonuclin-2* (*bnc2*) [33], which encodes a highly conserved zinc finger protein that may function as a transcription factor or in RNA processing [34]–[38]. In contrast to the wild-type, *bnc2* mutants exhibit far fewer hypodermal melanophores, xanthophores and iridophores and, consequently, lack body stripes and interstripes, though an apparently normal pigment pattern persists in the fins and in the scales (Figure 1A, 1B). During the larval-to-adult transformation of *bnc2* mutants, differentiated pigment cells of all three classes die at high frequency. Nevertheless, precursors of melanophores and xanthophores are abundant and widespread, suggesting late defects in their survival, terminal differentiation, or both. By contrast, iridophore precursors are markedly fewer, raising the possibility of additional defects in the earlier specification of this lineage. Genetic mosaic analyses showed that *bnc2* acts non-autonomously to the melanophore lineage and likely the other pigment cell classes as well. Consistent with this interpretation, *bnc2*+ cells are initially found along horizontal and vertical myosepta but are later widely dispersed, both in the hypodermis and epidermis, a distribution resembling that of fibromodulin-expressing fibroblasts (LP and DP, unpublished data) but distinct from that of pigment cells and their precursors.

Here, we investigated the mechanisms by which *bnc2* supports pigment cell development and the subsequent interactions between pigment cells during pigment pattern formation. We found that *bnc2* mutants have reduced expression of Csf1r ligands and the ligand of the Kit receptor tyrosine kinase, Kitlga, which is required for the migration, survival and differentiation of teleost melanophores as well as mammalian melanocytes [9], [39]–[44]. Although restoring Csf1 and Kitlga in *bnc2* mutants was sufficient to restore xanthophores and melanophores, these cells failed to organize into a normal striped pattern, indicating a requirement for additional factors or cell types. Because iridophores are deficient in *bnc2* mutants, we asked whether these cells might normally contribute to the formation of stripes and interstripes. We found that iridophores are the first adult pigment cells to develop, that they express Csf1, and that xanthophores localize in association with them. To test if interstripe iridophores contribute to pattern development, we ablated these cells in wild-type and mutant larvae, resulting in perturbations to stripes and interstripes and confirming roles for iridophores in stripe and interstripe development. Together, our analyses suggest a model in which *bnc2* supports the development and survival of melanophores, xanthophores and iridophores, and allows for subsequent interactions involving all three cell types. These results extend our understanding of environmental influences on pattern formation as well as pigment-cell autonomous patterning mechanisms.

## Results

### *bnc2-*dependent expression of Kitlga and Csf1 promotes melanophore and xanthophore development yet is insufficient for normal stripe patterning

The death of melanophores and xanthophores in *bnc2* mutants resembles the death of melanophores in mutants for *kita*, encoding a zebrafish Kit orthologue [41], and the death of xanthophores in *csf1r* mutants [24]. As *kita* and *csf1r* act autonomously to melanophore and xanthophore lineages [24], [41], respectively, whereas *bnc2* acts non-autonomously [33], we speculated that *bnc2* might contribute to the development and maintenance of melanophores and xanthophores by promoting expression of the receptor ligands, Kitlga and Csf1. Consistent with this idea, quantitative RT-PCR of isolated body skins (with attached pigment cells) revealed significantly reduced expression of *kitlga*, as well as the two Csf1-encoding loci, *csf1a* and *csf1b*, in *bnc2* mutants compared to the wild-type (Figure 1C). Quantitative RT-PCR comparisons of fins, in which melanophores and xanthophores persist in *bnc2* mutants, failed to reveal differences in *kitlga*, *csf1a* or *csf1b* expression compared to the wild type (all *P*>0.5; data not shown).

If *bnc2* acts through Kitlga and Csf1 to promote the development and survival of melanophores and xanthophores on the body, then restoring the expression of these ligands in the *bnc2* mutant should restore melanophores and xanthophores and possibly a striped pattern. To test this idea, we generated transgenic lines using the ubiquitous, heat-shock inducible promoter of *hsp70l* to express Kitlga, Csf1a, or Csf1b individually, as well as Kitlga simultaneously with either Csf1a or Csf1b.

**Figure 2:**
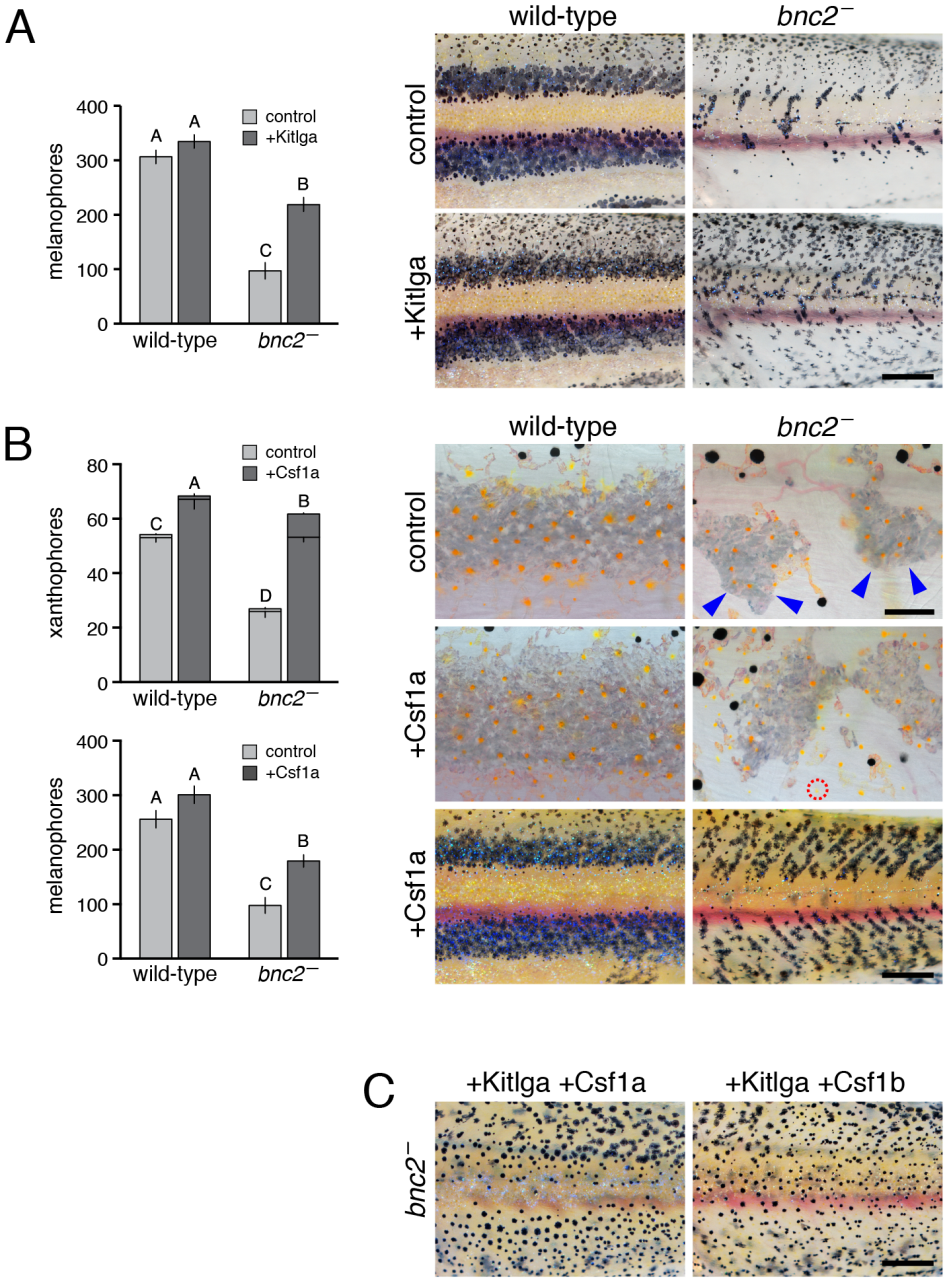
Re-expression of Kitlga, Csf1a, and Csf1b in *bnc2* mutants promoted melanophore and xanthophore development but was insufficient for stripe patterning. (A) Melanophore recovery following heat-shock induction of *Tg(hsp70l:kitlga)*. Although Kitlga expression increased melanophore numbers in *bnc2* mutant larvae, the restored melanophores failed to develop into stripes. Plots show means±SE with different letters above bars denoting means that differed significantly from one another in Tukey Kramer *post hoc* comparisons. All wild-type larvae are *bnc2*/+ siblings to *bnc2* mutants. Sample sizes: *bnc2*/+, *n* = 10; *bnc2*, *n* = 10, *bnc2*/+ *hsp70l:kitlga*, *n* = 14; *bnc2 hsp70l:kitlga*, *n* = 14. (B) Xanthophore and melanophore recovery following heat-shock induction of *Tg(hsp70l:csf1a)*. Upper plot, xanthophores were classed as either associated with iridophores (larger, lower segment of each bar), or not associated with iridophores (smaller, upper segment of each bar): total xanthophore numbers, including xanthophores not associated with iridophores were increased in *bnc2* mutants by Csf1a expression. Lower plot indicates that melanophore numbers were increased as well. Images show xanthophores (yellow–orange cells) over iridophores (patches of grey cells in this illumination, denoted by blue arrowheads in the *bnc2* mutant). Red dashed circle in *bnc2* mutant +Csf1 panel shows a xanthophore that has developed at a distance from iridophores. Lower magnification images (bottom) show typical patterns and the absence of organized stripes in the *bnc2* mutant after Csf1 expression, despite increased numbers of melanophores and xanthophores (compare to controls in A). Sample sizes: *bnc2*/+, *n* = 15; *bnc2*, *n* = 19, *bnc2*/+ *hsp70l:csf1a*, *n* = 19; *bnc2 hsp70l:csf1a*, *n* = 22. Results for *Tg(hsp70l:csf1b)* were equivalent (not shown; total sample size, *N* = 17). (C) Xanthophore and melanophore numbers were restored by heat shock induction of *Tg(hsp70l:kitlga-csf1a)* and *Tg(hsp70l:kitlga-csf1b)* yet stripes failed to form (total sample sizes, *N* = 7, 12, respectively). Scale bars: in (A) 500 µm for (A); in (B, upper) 80 µm for (B upper for images); in (B, lower) 500 µm for (B bottom 2 images); in (C) 500 µm for (C).

Restoration of Kitlga expression partially rescued melanophores in *bnc2* mutants but did not restore stripes (Figure 2A); this outcome was not unexpected given requirements for interactions between melanophores and xanthophores and the continued deficiency of the latter [23], [24], [26]. Restoration of Csf1a rescued xanthophores, and also increased melanophore numbers (Figure 2B). Despite the abundance of both cell types, normal stripe patterns again failed to develop, with melanophores and xanthophores ranging widely over the flank (Figure 2B). Similar outcomes were observed upon expressing Kitlga simultaneously with either Csf1a or Csf1b (Figure 2C), for Csf1b alone, and in genetic mosaics combining cells from Kitlga and Csf1a transgenic embryos (data not shown). Together, these findings support the idea that *bnc2*-dependent expression of Kitlga, Csf1a and Csf1b promotes the development and survival of hypodermal body melanophores and xanthophores, yet the presence of these cell types alone is insufficient for organizing a normal pattern of body stripes and interstripes.

### *bnc2*-dependent iridophores differentiate before melanophores and xanthophores and mark the prospective interstripe

The failure to recover a normal pigment pattern in *bnc2* mutants suggested that *bnc2* might contribute to interstripe and stripe development through another factor or cell type. We reasoned that such a role could be fulfilled by iridophores, which are dramatically fewer in *bnc2* mutants [33]. Consistent with this idea, residual xanthophores in the weak interstripe of *bnc2* mutants were found almost exclusively within patches of residual iridophores (compare images of xanthophores and iridophores between wild-type and *bnc2* mutant controls in Figure 2B).

**Figure 3:**
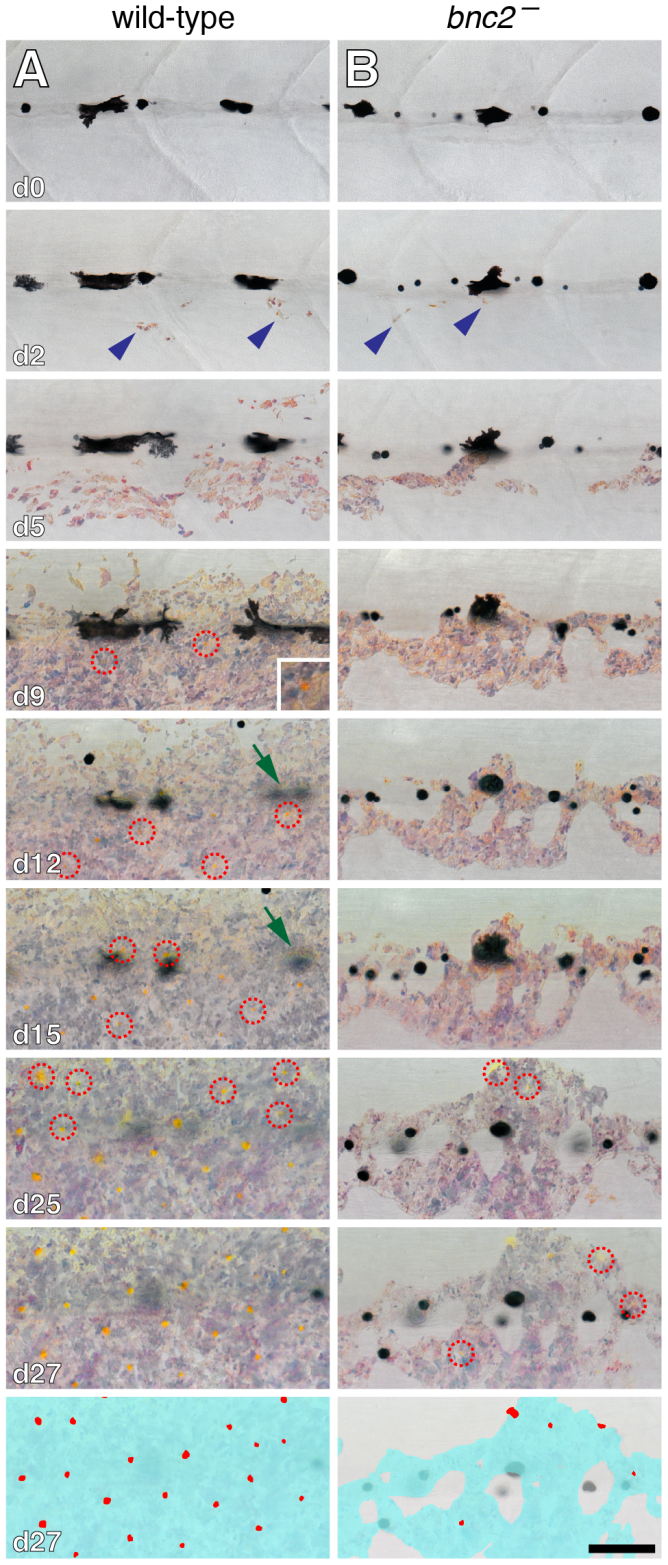
Interstripe xanthophores developed after iridophores in wild-type larvae and were further delayed in *bnc2* mutants. Shown are a representative wild-type (*bnc2*/+) larva (A) and a sibling *bnc2* mutant (B) imaged repeatedly over 27 d beginning at 6.0 SSL, just prior to the appearance of iridophores at the anteroposterior region imaged, dorsal to the anus. In both the wild-type and the *bnc2* mutant iridophores started to appear by day 2 of imaging (blue arrowheads). Xanthophores started to differentiate by day 9 of imaging in wild-type; newly arising xanthophores are indicated by red dashed circles. In contrast, xanthophores did not appear until day 25 of imaging in the *bnc2* mutant. As iridophores (and xanthophores) in the interstripe became more abundant, some early larval melanophores along the horizontal myoseptum disappeared from view (e.g., green arrows in A, d12 and d15). For easier visualization of melanophores and other cell type, fish were treated briefly with epinephrine immediately prior to imaging, which contracts melanosomes towards the cell body; the distribution of melanin thus indicates the centers of melanophores whereas processes extending out from the cell body are not visible. Bottom panels schematize the distribution of iridophores (light blue) and xanthophores (red) on the final day shown. Samples sizes for which complete image series were obtained were: *bnc2*, *n* = 4; *bnc2/+*, *n* = 6. Scale bar: in (B, d27) 80 µm for (A,B).

If iridophores contribute to patterning interstripe and stripe development, these cells should develop prior to xanthophores and melanophores. We confirmed this by repeated imaging of wild-type and *bnc2* mutant larvae, which showed that iridophores are the first adult pigment cell type to develop during the larval-to-adult transformation (Figure 3). Iridophores developed as early as 4.5 mm standardized standard length (SSL) [45] and were restricted initially to the prospective interstripe region anteriorly, then developed in progressively more posterior regions. In contrast, the first melanophores and xanthophores differentiated later at ∼6.0 SSL and ∼6.5 SSL, respectively. In *bnc2* mutants, xanthophore development was significantly delayed (*F*_1,5_ = 383.8, *P*<0.001), typically occurring at ∼7.5 SSL. The time and place of iridophore development relative to xanthophores and melanophores make iridophores a good candidate for contributing to interstripe location and orientation, and potentially later stripe patterning and maintenance.

### Iridophores influence the localization of xanthophores and melanophores during interstripe and stripe development

**Figure 4:**
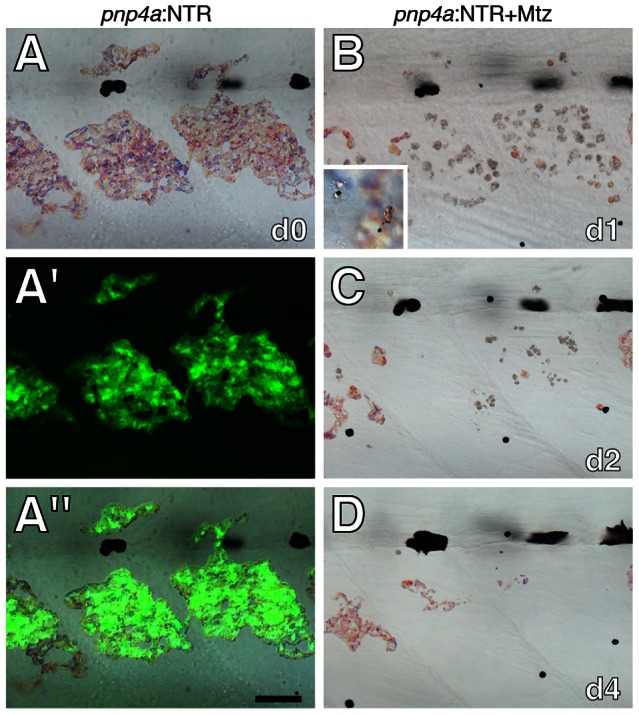
Ablation of iridophores by Mtz treatment of fish injected with *pnp4a*:NTR. (A) Iridophores in a wild-type larva (6.5 SSL) were marked by Venus fluorescence following injection of *pnp4a*:nlsVenus-V2a-NTR plasmid as the 1-cell stage, as shown in bright-field (A), fluorescence (A′) and merged (A″) views. (B–D) The same larva following Mtz treatment exhibited fewer, rounded iridophores that were progressively lost over several days. Inset in B shows reflecting-platelet containing extrusion bodies at the surface of the epidermis. Scale bar: in (A″) 60 µm for (A–D).

To test whether iridophores contribute to specifying the location of interstripe xanthophores, we sought to ablate iridophores specifically and autonomously. To this end, we isolated a 3.2 kb fragment upstream from the transcriptional start site of the iridophore marker gene *purine nucleoside phosphorylase 4a (pnp4a)* [11], [33] that drives iridophore-specific transgene expression (Figure 4A). We used this element to express bacterial nitroreductase (NTR), which converts metronidazole (Mtz) into toxic metabolites that kill cells without bystander effects, even amongst cells that are coupled gap-junctionally [46]–[50]. We injected embryos with this *pnp4a*:NTR construct at the one-cell stage and then treated these genetically mosaic larvae with Mtz at stages when adult iridophores first develop in the prospective interstripe. Iridophores were lost over several days and reflecting-platelet containing fragments were identified in typical “extrusion bodies” [33], [41], [42] at the surface of the epidermis (Figure 4B, 4C, 4D). In contrast to transient, F0-injected transgenic larvae, it was not possible to ablate iridophores in stable *pnp4a*:NTR lines, presumably because of reduced transgene copy numbers. Thus, all subsequent analyses used genetically mosaic F0 larvae with repeated Mtz treatments.

**Figure 5:**
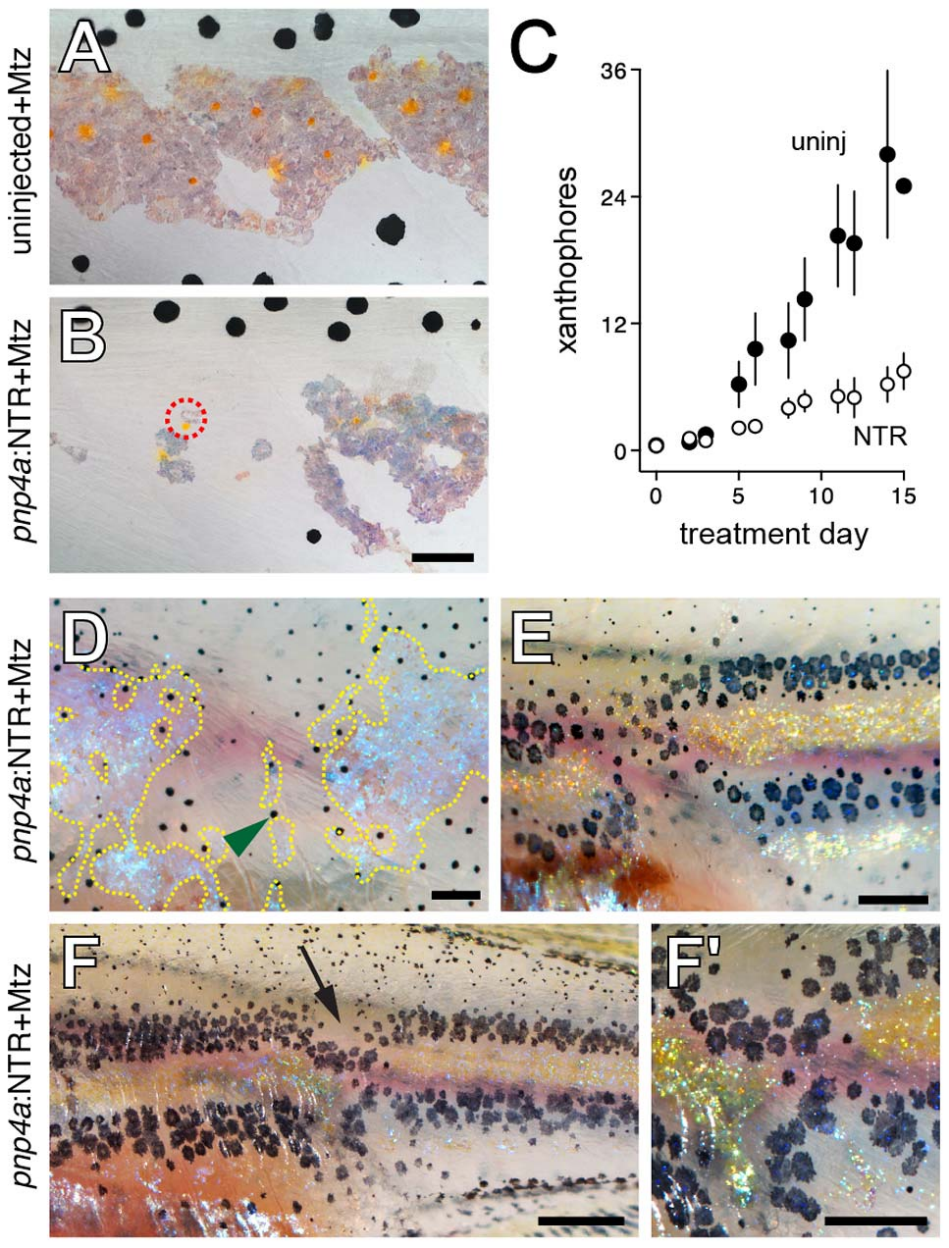
Iridophore ablation perturbed xanthophore and melanophore patterning. (A, B) Wild-type siblings that were either not injected (A) or injected (B) with *pnp4a*:NTR plasmid and then treated with Mtz beginning at 5 SSL, prior to the the onset of xanthophore differentiation. Controls (A) exhibited normal interstripe iridophores and xanthophores whereas iridophore-ablated individuals developed xanthophores primarily in association with residual iridophores (e.g., dashed red circle in B). (C) Numbers of xanthophores (means±SE) in stage-matched siblings treated with Mtz that were either uninjected or injected with *pnp4a*:NTR plasmid. Xanthophore numbers did not differ between groups at the onset of the experiment but iridophore-ablated individuals showed an increasingly severe xanthophore deficiency compared to uninjected larvae as the experiment proceeded (genotype×day interaction, *F*_1,10_ = 2.7, *P*<0.005; initial sample sizes: uninjected, *n* = 13; *pnp4*:NTR, *n* = 13). During later development, new xanthophores ultimately developed more broadly over the flank and in association with regenerating iridophores; iridophore ablations after xanthophores had differentiated typically did not affect these cells (not shown). (D–F) Examples of larvae (9.5 SSL) exhibiting melanophore patterning defects following earlier iridophore ablations (started at 6.0 SSL). Melanophores have colonized regions from which iridophores were ablated, though a few regenerative or persisting iridophore remained. In the lighting used here, iridophores are blue or gold iridescent. (D) Melanophores occupy a region from which iridophores were ablated (residual or regenerated iridophores outlined by dashed yellow lines). Green arrowhead, one of several melanophores localized adjacent to remaining iridophores. Fish shown in A, C and D were treated with epinephrine prior to imaging. (E) Melanophore stripes are broken at site of iridophore ablation and melanophores appear to “wrap around” residual interstripe iridophores on either side of the ablation. (F) In another individual, melanophores stripes are constricted where iridophores have been ablated (arrow). Close-up in F′. Fish in E and F were not treated with epinephrine, so that melanin reveals peripheral processes of melanophores. Most small melanophores in dorsal regions are associated with developing scales and will not contribute to the stripe pattern [45]. (Total sample size, *N* = 40.) Scale bars: in (B) 60 µm for (A,B); in (D) 200 µm for (D); in (E) 500 µm for (E); in (F) 100 µm for (F); in (F′) 60 µm for (F′).

Ablation of interstripe iridophores prior to xanthophore development resulted in fewer xanthophores in regions from which iridophores were lost (Figure 5A, 5B, 5C), although both iridophores and xanthophores were recovered gradually during later development. Ablations of interstripe iridophores after xanthophores had developed typically did not affect xanthophore survival or patterning (data not shown).

Because interactions between xanthophores and melanophores contribute to organizing melanophore stripes, we anticipated that iridophore ablation and delayed xanthophore development could perturb melanophore patterning as well. Consistent with this prediction, we observed more melanophores in interstripe regions where iridophores (and xanthophores) had been depleted; nevertheless, melanophores occupying these regions were frequently found adjacent to residual or regenerated iridophores (Figure 5D, 5E, 5F).

### Iridophores express Csf1

**Figure 6:**
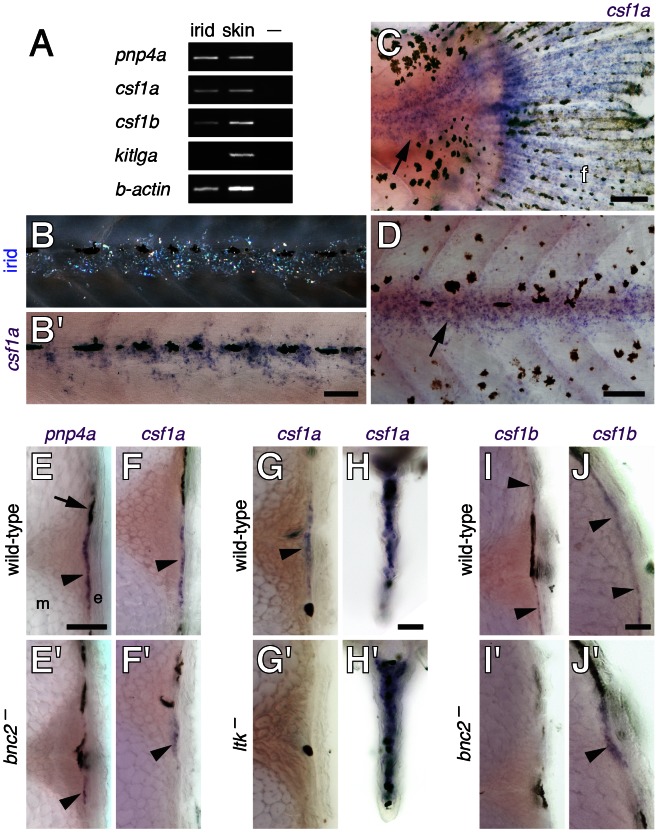
*csf1a* and *csf1b* were expressed by interstripe iridophores as well as hypodermal and fin cells. (A) RT-PCR of isolated iridophores (irid) and skin containing pigment cells for the iridophore marker *pnp4a* as well as *csf1a*, *csf1b* and *kitlga*. –, no template control. See text for details. (B) A larva (∼6 SSL) imaged to show iridophores prior to fixation (upper) and after whole-mount staining for *csf1a* transcript. Not all iridophore reflecting platelets are visible and platelets that are apparent may not precisely delineate cell bodies and processes. (C,D) Whole-mount larvae (∼8.5 SSL) stained for *csf1a* transcript. (C) *csf1a* was expressed in the posterior trunk at the base of the caudal fin (arrow) where a patch of posterior iridophores develops [45] and also within the fin (f). (D) *csf1a* staining near the horizontal myoseptum (arrow). (E–J) In situ hybridizations on vibratome cross-sections through the midtrunk (∼7 SSL). (E,E′) *pnp4a* staining indicated iridophore locations (arrowheads) within the hypodermis of wild-type (*bnc2*/+) larvae (E) and revealed fewer of these cells in *bnc2* mutants (E′). Arrow, melanophore. (F,F′) *csf1a* staining (arrowheads) was reduced in *bnc2* mutants. (G–H) Staining for *csf1a* in wild-type (*ltk*/+) and *ltk* mutants, which lack iridophores. (G,G′) *csf1a* staining was absent in *ltk* mutants at the location where iridophores are found in the wild-type (arrowhead). (H,H′) In the fins, however, iridophore-independent *csf1a* expression was present in both wild-type and *ltk* mutant larvae. (I–J) *csf1b* expression was at the limit of detection by in situ hybridization. (I,I′) Along the lateral trunk, *csf1b* transcript (arrowheads) was evident in wild-type larvae, representing either hypodermal cells, iridophores or both, but transcript was not apparent in *bnc2* mutant sections stained for equivalent times. (J,J′) Along the dorsal trunk, *csf1b* transcripts (arrowheads) were evident in both wild-type and *bnc2* mutants. Scale bars: in (B) 60 µm for (B); in (C) 100 µm for (C); in (D) 100 µm for (D); in (E) 80 µm for (E,E′,F,F′,G,G′,I,I′), in (H) 80 µm for (H,H′); in (J) 20 µm for (J,J′).

Given the dependence of Csf1 expression (Figure 1) and iridophore development on *bnc2* (Figure 3) [33], the requirement of xanthophores for signaling through Csf1r [22], [24], and the dependence of xanthophores on iridophores (above), we hypothesized that iridophores supply a localized source of Csf1 to promote xanthophore development in the interstripe. We confirmed that *csf1r* is expressed by xanthophores during the larval-to-adult transformation using a transgenic reporter line derived from a bacterial artificial chromosome containing the *csf1r* locus (Figure S1) [51]. To test if interstripe iridophores express *csf1a* and *csf1b*, we first used RT-PCR, which detected transcripts for both loci in iridophores isolated individually (Figure 6A). By in situ hybridization, we found *csf1a* transcripts in hypodermal cells including cells likely to be iridophores according to their positions before and after in situ hybridization, and their locations at the base of the caudal fin and along the horizontal myoseptum, where iridophores develop (Figure 6B, 6C, 6D). In cross-sections, *csf1a* transcript was detectable in the hypodermis where iridophores are found, as revealed by expression of *pnp4a* [11], [33] (Figure 6E, 6F). In contrast to wild-type larvae, far fewer cells stained for *pnp4a* and *csf1a* in the prospective interstripe region of *bnc2* mutants. To further test the correspondence of *csf1a* expression and iridophores we examined the iridophore-free mutant of *leucocyte tyrosine kinase* (*ltk*), which is expressed by iridophores and required for their development [52]. *ltk* mutants lacked *csf1a* expression where iridophores are found normally in wild-type larvae (Figure 6G, 6G′). We also observed strong, iridophore-independent expression of *csf1a* in fins of wild-type and *ltk* mutants (Figure 6H, 6H′). *csf1b* was expressed similarly to *csf1a* by in situ hybridization and was also detectable in a population of dorsal hypodermal cells in both wild-type and *bnc2* mutants. Together, these analyses indicate that iridophores express Csf1, and do so at a time and place that marks the prospective interstripe, though additional cell types express these ligands as well.

### Localized expression of Csf1 promotes regionally specific xanthophore development

**Figure 7:**
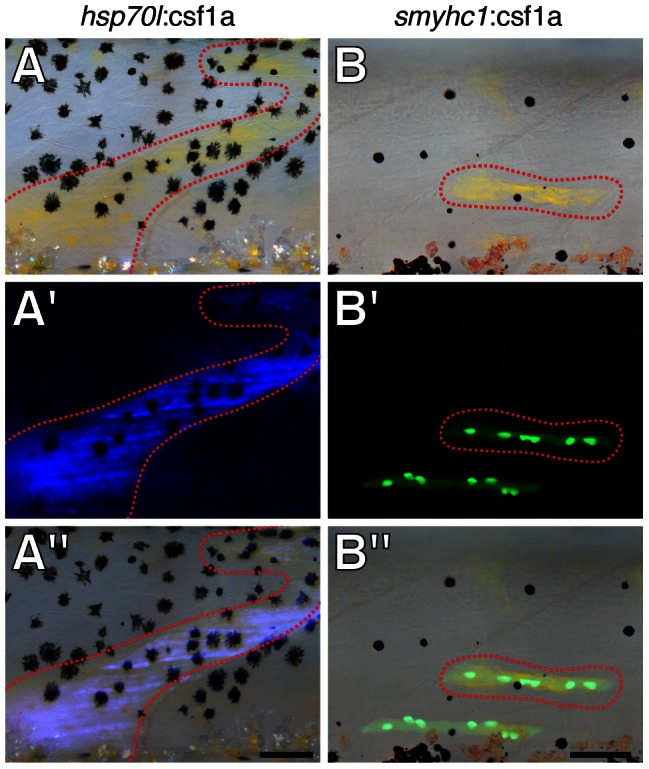
Localized Csf1 expression directed xanthophore development. (A) Ectopic xanthophores (red dashed line) developed over the dorsal myotome in association with Csf1a-expressing cells transplanted from a wild-type, *Tg(hsp70l:csf1a-IRES-nlsCFP)* donor to a *bnc2* mutant host. Larva shown at 7.9 SSL. (A′) Nuclear CFP expression in the myotome. (A″) Merge. (B) Ectopic xanthophores in a wild-type larva developed over the dorsal myotome in association with a slow muscle fiber of the myotome expressing Csf1a from plasmid *smyhc1*:TetGBD-TREtightBactinTRX:nlsVenus-V2a-csf1a. Larva shown at 7.5 SSL. (B′) Nuclear Venus expression. (B″) Merge. (Sample sizes: *hsp70l*, *n* = 8; *smyhc1*, *n* = 10.) Scale bars: in (A″) 100 µm for (A); in (B) 100 µm for (B).

If Csf1 expressed by early interstripe iridophores provides a spatial cue for xanthophores, we reasoned that ectopic expression of Csf1 should result in ectopic xanthophore development. To test this possibility we transplanted cells at the blastula stage from *bnc2* mutant embryos transgenic for *hsp70l*:csf1a to *bnc2*/+ or *bnc2* hosts and then induced mosaic expression of Csf1a by heat shock. We additionally expressed Csf1a in a temporally controlled manner within the myotome adjacent to the hypodermis: we identified a 2.2 kb region upstream of *slow myosin heavy chain 1* (*smyhc1*) that drives expression in superficial slow muscle fibers and used this in a TetA-GBD [53] transgene to express Csf1a in these cells specifically during the larval-to-adult transformation. Using both paradigms to induce Csf1a outside of the developing interstripe, we observed corresponding patches of ectopic xanthophores in both *bnc2*/+ and *bnc2* mutant siblings (Figure 7). These findings, and analyses of *csf1a* and *csf1b* expression, support a model in which interstripe iridophores provide a localized source of these ligands that contributes to specifying the position of interstripe xanthophores.

### Iridophores influence melanophore patterning independently of xanthophores

**Figure 8:**
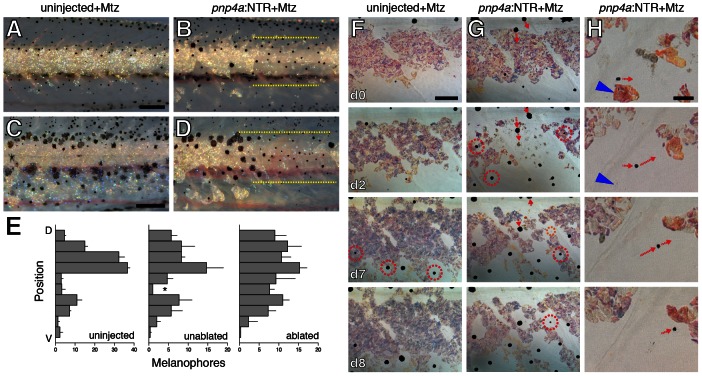
Iridophores influenced melanophore pattern in xanthophore-deficient *csf1r* mutants. (A,B) In stage-matched siblings treated with Mtz, a region on the tail from which iridophores have been ablated (dashed yellow lines in B) exhibits more melanophores than the corresponding region of the control larva (shown here at ∼8.5 SSL). Both larvae were treated with epinephrine immediately before imaging. (C,D) Iridophore ablation at the mid-trunk region (dashed yellow lines in D) likewise resulted in increased numbers of melanophores compared to stage-matched control (C)(shown here at ∼10.2 SSL). Note that some iridophores have regenerated within previously ablated regions and that melanophores are present at the left edge of the ablated region, adjacent to remaining interstripe iridophores. Larvae in these images were not treated with epinephrine. (E) Quantification of melanophore distributions within dorsal–ventral regions of the flank for larvae that were uninjected but treated with Mtz (left) and for regions of injected, Mtz-treated larvae from which iridophores were unablated (middle) or ablated (right). Plots show means±SE within each region. Asterisk denotes the residual interstripe in *csf1r* mutants, where melanophore numbers differed significantly between unablated and ablated regions (paired *t* = 5.6, d.f. = 2, *P*<0.05). (F,G) Details showing melanophore behaviors in an uninjected control larva (F) and an injected larva (G) in the region of iridophore ablation. Day 0 panels show initial distribution of iridophores and melanophores, prior to Mtz treatment (7.0 SSL). Following iridophore ablation (G), some melanophores moved short distances ventrally (red arrows at d0 and d2 show starting and stopping positions of two melanophores). Melanophores also differentiated within the ablated region (dashed red circles in G, d2); dashed orange circle in G, d7 shows a lightly melanized cell just beneath the surface of the myotome that emerges within the skin by d8. In unablated individuals (F), melanophores typically differentiated further ventrally at sites lacking iridophores (an exception is the left-most melanophore that appeared at d7). Also see Figure S3C. (H) Detail from another individual showing a lightly melanized cell initially near an iridophore that was ablated (blue arrowhead); the melanophore subsequently translocated to settle adjacent to another iridophore. All larvae in F–H were treated with epinephrine. (Total sample size, *N* = 55.) Scale bars: in (A) 200 µm for (A,C); in (B) 400 µm for (D); in (F, d0) 80 µm for (F,G); in (H, d0) 20 µm for (H).

Because xanthophores contribute to melanophore stripe organization [23], [24], [26], the mis-patterning of melanophores following iridophore ablation could simply reflect perturbations to the distribution of xanthophores. Yet, iridophores also might influence melanophores independently of xanthophores. To test this possibility, we ablated iridophores in *csf1r* mutant larvae. These mutants exhibit a few very lightly pigmented xanthophores limited to the immediate vicinity of the horizontal myoseptum but lack xanthophores in the more ventral interstripe region and elsewhere (Figure S2A, S2B) [22], [23], [54]. Although stripes in csf1r mutants are disorganized and melanophores initially differentiate more widely over the flank than in wild-type larvae [22], quantitative analyses of final melanophore distributions in unmanipulated *csf1r* mutants revealed a residual stripe pattern in which melanophores tended to be dorsal or ventral to where the interstripe would form normally (Figure 8A, 8C, 8E). At later stages, melanophores tended to be situated close to, but not directly over, iridophores, and iridophores were more widely distributed than in the wild-type (Figure S2C). In *csf1r* mutants in which iridophores had been ablated, however, melanophores were more likely to occur in the middle of the flank where iridophores had been lost (Figure 8B, 8D, 8E). Repeated imaging of individual larvae showed that melanophores both migrated to, and differentiated in, regions where iridophores had been ablated; once in these regions, melanophores often settled adjacent to residual iridophores (Figure 8F, 8G, 8H). Together, these observations suggest that iridophores can influence melanophore patterning independently of interactions between xanthophores and melanophores. Although *kitlga* is a good candidate for contributing to an interaction between iridophores and melanophores, *kitlga* expression by iridophores was not detected by RT-PCR or in situ hybridization (Figure 6A and data not shown).

### Melanophore and xanthophore patterning are defective in additional iridophore-deficient mutant backgrounds

**Figure 9:**
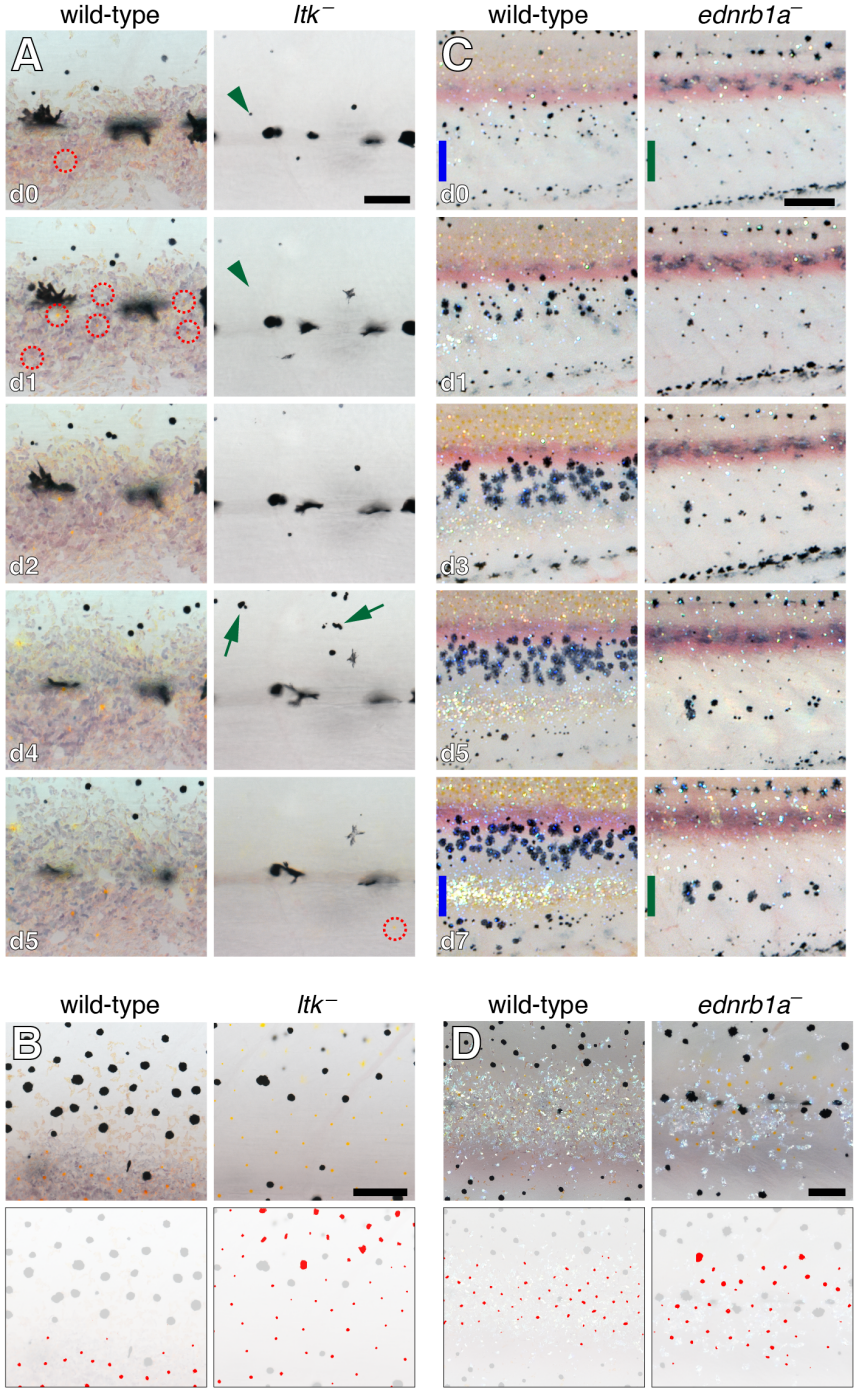
Melanophore and xanthophore development is disrupted in additional iridophore-deficient mutants. (A) Comparison of xanthophore and melanophore development in wild-type and *ltk* mutants. Shown are details at the horizontal myoseptum from larger images of representative wild-type (*ltk*/+) and *ltk* mutant, stage-matched siblings imaged daily (beginning at 6 SSL). In the wild-type, nearly all melanophores persisted through the image series. A xanthophore had already developed at the onset of imaging (day 0, red dashed circle), and additional xanthophores differentiated shortly thereafter. In the *ltk* mutant, however, melanophores were frequently lost between days (green arrowheads) and melanin-containing debris and extrusion bodies were often apparent (green arrows). Unlike the wild-type, no xanthophores differentiated until day 5 of imaging. (B) During later development (9.6 SSL), xanthophores were confined principally to the interstripe region of the wild-type whereas xanthophore developed widely over the flank in the *ltk* mutant. The horizontal myoseptum lies at the lower edge of both images. Lower panels show positions of xanthophores in red. (C) Comparison of wild-type and *ednrb1a* mutant. Shown are ventral flanks of representative stage-matched, sibling wild-type (*ednrb1a*/+) and *ednrb1a* mutant larvae imaged daily (8.8–10 SSL). At the onset of imaging, wild-type melanophores are largely absent from a region where the second interstripe will form by day 7 of imaging (blue bars). In *ednrb1a* mutants, however, melanophores are relatively uniformly distributed in this region at the onset of imaging, and, by day 7 of imaging, formed clusters where the second interstripe would normally form (green bars). Images shown were rescaled to control for growth. (D) Closeups showing reduced iridophores in *ednrb1a* mutant compared to wild-type (9.0 SSL) as well as wider distribution of xanthophores. Fish in A, B and D were treated briefly with epinephrine prior to imaging. Sample sizes for which complete image series were obtained were: *ltk*, *n* = 6; *ltk/+*, *n* = 5; *ednrb1a*, *n* = 4; *ednrb1a/+*, *n* = 5. Scale bars: in (A, d0) 60 µm for (A); in (B) 100 µm for (B); in (C, d0) 200 µm for (C, d0); in (D) 100 µm for (D).

To further test inferences from cell ablation studies, we examined melanophore and xanthophore patterning in additional mutant backgrounds, *ltk*, described above, and *endothelin receptor b1a* (*ednrb1a*). *ltk* mutants lack iridophores and repeated imaging of individual larvae revealed increased frequencies of melanophore death, as well as delays in xanthophore differentiation by an average of 6±1 d (paired *t* = 6, *P*<0.05) as compared to stage-matched wild-type siblings (Figure 9A). When xanthophores did develop they did so widely over the flank, rather than being restricted to the interstripe region (Figure 9B).

*ednrb1a* is expressed in precursors to all three pigment cell classes and is maintained at high levels in iridophores [55]. *ednrb1a* mutants exhibit severely reduced numbers of iridophores (Figure 9D). Although adults exhibit a dorsal melanophore stripe and ventral melanophore spots, examination of pattern development in daily image series showed that ventral spots arise further ventrally than the normal location of the ventral stripe, being localized instead to the site of the second ventral interstripe (Figure 9C). Together these observations indicate that melanophore and xanthophore patterning are disrupted in two additional iridophore-deficient mutants, consistent with roles for iridophores in promoting normal stripe and interstripe development.

## Discussion

**Figure 10:**
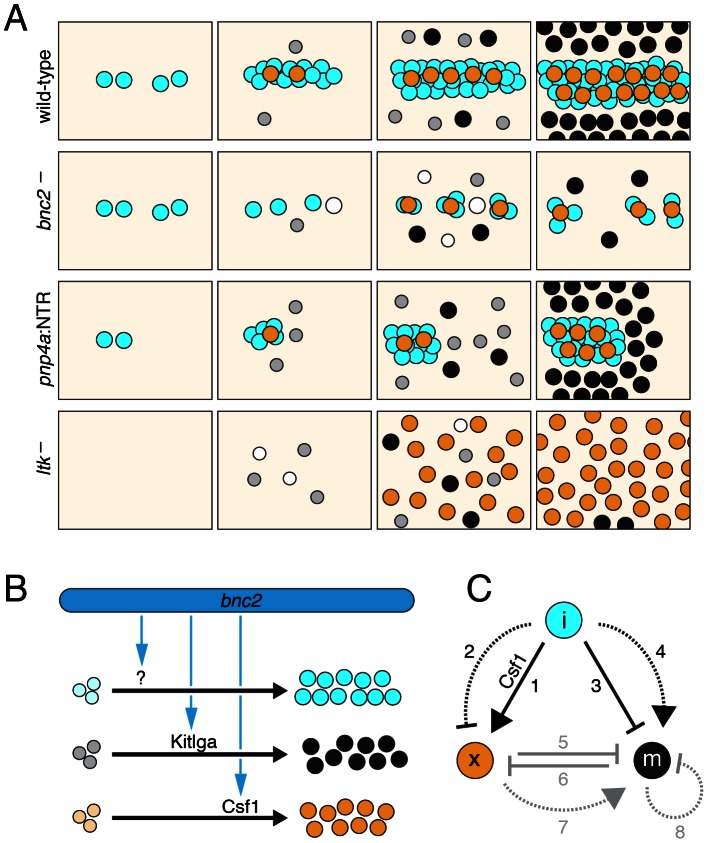
Summary of results and model for stripe and interstripe patterning in zebrafish. (A) Development of pigment pattern phenotypes in wild-type, *bnc2* mutants, iridophore-ablated larvae (*pnp4a*:NTR), and *ltk* mutants. Blue circles, iridophores; orange circles, xanthophores; grey and black circles, melanophores. In *bnc2* mutants, there are fewer iridophores and increased rate of cell death (open circles) amongst all three pigment cell classes. Xanthophores are restricted to the vicinity of iridophores. In iridophore-ablated larvae, melanophores localize where iridophores have been lost but also organize adjacent to residual iridophore patches. In *ltk* mutants, iridophores are missing, melanophores tend to die, and xanthophores develop both later and over a wider area than in wild-type larvae. (B) An unknown, *bnc2*-dependent factor expands an initial population of iridophores, whereas *bnc2*-dependent Kitlga and Csf1 support the expansion of melanophore and xanthophore populations. (C) Hypothesized interactions amongst pigment cell classes. Black lines, suggested by this study; grey lines, suggested previously [23], [24], [26]. Solid lines, short-range interactions; dotted lines; longer-range interactions. Iridophores promote xanthophore localization to the prospective interstripe at short-range through Csf1 (interaction #1), and are hypothesized to repress xanthophore development at a distance (#2). Iridophores also affect melanophores, which are inhibited from localizing at sites already occupied by iridophores (#3), and instead differentiate or localize nearby (#4). Once melanophores and xanthophores have developed, these cells exhibit mutual, short-range inhibitory interactions that affect localization, survival or both (#5, #6); xanthophores also promote melanophore survival at a distance (#7) and melanophores repress the development of other melanophores at a distance (#8) [26]. See main text for additional details.

Our analyses together with previous studies suggest a model for adult body stripe and interstripe development in zebrafish (Figure 10). At the onset of adult pigment pattern formation, iridophores begin to differentiate in the prospective interstripe region and the expansion of this population depends on *bnc2*. Melanophores and xanthophores then start to differentiate, supported by *bnc2*-dependent Kitlga and Csf1, respectively. Melanophores avoid settling in the interstripe region in part owing to short-range inhibitory interactions with iridophores, whereas xanthophores differentiate specifically in the interstripe, receiving Csf1 both from the skin and from iridophores already there. Subsequently, interactions among all three classes of pigment cells contribute to organizing the definitive pattern of stripes and interstripes.

Previous analyses of adult pigment pattern formation in zebrafish highlighted the importance of interactions between melanophores and xanthophores [23], [24] and a combination of short-range and long-range interactions between these cell types is consistent with a Turing mechanism of pattern formation or maintenance [26], [27]. Nevertheless, one might anticipate roles for additional cues in specifying stripe position or orientation. For example in studies using a temperature-sensitive allele of *csf1r*, the orientation of stripes in the fin was randomized when xanthophores developed only at late stages [24], suggesting that cues required for orienting stripes during development either were not present, or not recognized, at later stages. Similarly in this study, the recovery of widespread melanophores and xanthophores in *bnc2* mutants was insufficient for stripe formation on the body. This observation suggested that additional factors specify the location and orientation of stripes and interstripes, and support melanophores and xanthophores during pattern formation.

This study indicates that iridophores contribute to adult pigment pattern formation, with several lines of evidence implicating interstripe iridophores in the development of interstripe xanthophores. First, image analyses showed that iridophores are the first adult pigment cells to develop, and do so at the interstripe. Second, Csf1r signaling is necessary for xanthophore development [22], [24] and we found that interstripe iridophores express *csf1a* and *csf1b* whereas xanthophores express *csf1r*. Third, misexpressing Csf1 resulted in the development of ectopic xanthophores, indicating this pathway can promote xanthophore localization. Fourth, xanthophore development was delayed when iridophores were ablated transgenically and in the *bnc2* mutant, which has a severe iridophore deficiency. Fifth, the few xanthophores that do develop in *bnc2* mutants were associated exclusively with the few residual iridophores. From these observations we suggest that iridophores promote the timely appearance of xanthophores within the interstripe (Figure 10C, interaction #1), thereby positioning xanthophores to interact with melanophores during the subsequent patterning of dorsal and ventral stripes.

Our finding that xanthophore development is delayed in iridophore-deficient *ltk* mutants is consistent with these inferences. That xanthophores ultimately differentiated in these mutants presumably reflects the persistence of iridophore-independent sources of Csf1 that are not present or not sufficient for xanthophore development in *bnc2* mutants. Interestingly, when xanthophores did develop in *ltk* mutants, they did so more widely over the flank than in the wild-type, in which xanthophores were restricted to the interstripe. A similar restriction of xanthophores to the vicinity of interstripe iridophores has been reported for *mitfa* mutants, which retain iridophores yet lack melanophores [23]. These observations raise the possibility that iridophores both promote xanthophore development at short-range and repress xanthophore development at long-range (Figure 10C, interaction #2), though we cannot yet exclude other explanations for this phenomenon.

Our analyses also suggest roles for iridophores in melanophore development and patterning. Our finding that melanophores localized to regions from which iridophores had been ablated could reflect a delay in the development of xanthophores and the inhibitory effects that xanthophores have on melanophore localization [26]. Although this may have contributed to the mis-patterning of melanophores, our finding that iridophore ablation perturbs melanophore patterning even in xanthophore-deficient *csf1r* mutants suggests that iridophores also influence melanophores independently of xanthophores. Melanophores frequently migrated to, or differentiated within, iridophore-free sites; melanophore centers (as indicated by melanosomes contracted by epinephrine) rarely overlapped with iridophores, yet melanophores often settled adjacent to iridophores. These observations are consistent with a very short-range inhibitory effect of iridophores on melanophore localization (Figure 10C, interaction #3), as might occur if the two cell types compete for a common substrate, as well as a longer-range attractive or stimulatory effect of iridophores on melanophores (Figure 10C, interaction #4). Our findings of increased melanophore death in *ltk* mutants, and the increased death of *mitfa*:GFP+ cells [16] as well as mis-patterning of melanophores in *ednrb1a* mutants, are likewise consistent with a model in which iridophores influence melanophores. Finally, we note that our examination of *csf1r* mutants revealed iridophores to be more widespread in this xanthophore-deficient background than in the wild-type, raising the possibility that xanthophores interact reciprocally with iridophores as well as melanophores. A definitive test of the interactions hypothesized in Figure 10C will await the elucidation of molecular mechanisms underlying these various pattern-forming events.

In addition to interactions among pigment cells, our study provides new insights into roles for *bnc2* in pigment pattern development. Expression analyses and rescue experiments suggested that *bnc2* promotes the development and survival of melanophores and xanthophores by ensuring adequate expression of *kitlga*, *csf1a*, and *csf1b* (Figure 10B). These observations are consistent with previously known roles for Kit ligand [16], [40]–[44], [56]–[58] and Csf1 [22]–[24], [59], and identify a novel role for Bnc2 in regulating the expression of these genes. It will be interesting to learn if Bnc2 has similar functions in providing trophic support to other stem-cell derived lineages as this locus is also expressed in the ovary, central nervous system, and skeleton [33]. Indeed, zebrafish bnc2 mutant females are infertile and human *BNC2* variants are associated with ovarian cancer predisposition [60]; potential defects in other systems have yet to be ascertained.

At least two aspects of *bnc2* function remain ambiguous. First, although it is clear that *bnc2*-dependent iridophores provide one source of Csf1 to developing xanthophores, *csf1a* and *csf1b* are also expressed more broadly, whereas *kitlga* is expressed in skin, and it has not yet been possible to establish whether *bnc2*+ cells express these factors themselves, or induce other cells to do so. The development of transgenic reporters for all of these loci will address this issue definitively. Second, iridophores are the most severely affected cell type in *bnc2* mutants [33] yet the mechanism by which *bnc2* promotes iridophore development remains unknown. The distribution of *bnc2*+ cells [33] does not perfectly mark the prospective interstripe so it seems likely that other factors specify where iridophores will develop, with *bnc2*+ cells promoting the expansion of the interstripe iridophore population once it has been established. It will be interesting to learn which *bnc2*-dependent and *bnc2*-independent factors are required for iridophore development and whether manipulation of these factors is sufficient to alter the location or orientation of stripes and interstripes. Finally, the continued high expression of Csf1 and Kitlga in the fins of *bnc2* mutants seems likely to explain the persistence of stripes and interstripes at this location; why fin melanophores and xanthophores can organize into stripes in the absence of *bnc2* activity, whereas body melanophores cannot awaits further investigation.

Several studies have highlighted the pigment-cell autonomous nature of pattern-generating mechanisms in zebrafish. Our study suggests two extensions to this paradigm. First, environmental factors are required to support pigment cells during pattern formation and are likely to provide cues that bias the initial development of pigment cells (e.g., the first interstripe iridophores) to specific regions, thereby influencing the subsequent locations and orientations of stripes and interstripes. Second, interactions among pigment cells appear to involve all three major classes. Analyses presented here support a model in which iridophores exert positive and negative effects on both xanthophores and melanophores and we can imagine that additional interactions will be identified as well. In this regard, it will be interesting to learn whether pigment pattern formation occurs through additional dimensions of Turing-like interactions. Because interactions amongst more than two cell types are not readily accommodated by existing frameworks for describing local self-activation with lateral inhibition mathematically, additional theoretical effort will be needed to capture biological complexity involving multiple cell types and multiple, reciprocal interactions. Finally, we envisage that evolutionary changes in factors both non-autonomous and autonomous to pigment cell lineages are likely to have contributed to the extraordinary diversification of pigment patterns among ectothermic vertebrates; it will be exciting to discover what general themes emerge as mechanisms of pigment pattern formation are elucidated in other species.

## Materials and Methods

### Ethics statement

All animal studies were conducted in accordance with regulations of the University of Washington and the United States Department of Health and Human Services, and received the approval of the Institutional Animal Care and Use Committee of the University of Washington.

### Fish stocks, staging, transgenes, cell-transplantation, and rearing conditions

Wild-type stock fish, WT(WA), were generated by crosses between the inbred genetic strains AB^wp^ and wik or the progeny of such crosses. Mutants were presumptive null alleles *bnc2^utr16e1^* [33], *csf1r^j4e1^* and *csf1r^j4blue^* [22], and *mitfa^w2^* [61], as well as hypomorphic alleles *ltk^j9s1^* [52] and *ednrb1a^b140^* [55]. Transgenic lines were *Tg(hsp70l:kitlga)^wp.r.t2^*, *Tg(hsp70l:csf1a-IRES-nlsCFP)^wp.r.t4^*; *Tg(hsp70l:csf1b-IRES-nlsCFP)^wp.r.t5^*, *Tg(hsp70l:kitlga-V2a-csf1a-IRES-nlsCFP)^wp.r.t6^*, *Tg(hsp70l:kitlga-V2a-csf1b-IRES-nlsCFP)^wp.r.t7^* and *Tg(csf1r:Gal4.VP16)^i186^*; *Tg(UAS-E1b:nfsB.mCherry)^i149^* [51]. Post-embryonic stages are reported as standardized standard length (SSL) measurements following [45]; SSL provides a more accurate representation of stages than days post-fertilization.

Transgenes were assembled by Gateway cloning of entry plasmids into pDest vectors containing *Tol2* repeats for efficient genomic integration [62], [63]. For expressing NTR in iridophores, we cloned the upstream region of *pnp4a* [11], [33] using primers (forward, reverse): CCTGGGTTTTTGCCATTCTTTAGG, GAATGAGAGAGCAGCTCTTTCC. To express Csf1a in slow muscle cells of the myotome we cloned a region upstream of *smyhc1* using primers (forward, reverse): AACAAGAAGAGCAAGAGGTTGAGGT, CAGATGAACAAACTTATAAATATAATGTGCTTCTCT. Microinjection of plasmids and *Tol2* mRNA used standard methods. Cell transplantation followed [33].

Fish stocks were reared in standard conditions at 28.5°C 14L:10D. For transgene inductions using *hsp70l* promoters, fish were heat-shocked at 38°C twice daily for 1 h beginning when fish had reached 8.5 SSL and extending for period 2–4 weeks. For fish injected with plasmid *smyhc1*:TetGBD-TREtightBactinTRX:nlsVenus-V2a-csf1a, induction with dexamethasone and doxycycline followed [53]. For ablating iridophores in larvae mosaic for plasmid *pnp4a*:nlsVenus-V2a-NTR, larvae were incubated overnight in 10 mM Mtz. For time series of individual ablations in wild-type and *csf1r* mutants, larvae were allowed to recover one night prior to imaging. Fish were then imaged a second day and treated with Mtz again that evening. Repeated treatments were required to repress iridophore regeneration, though in many cases, iridophores eventually recovered. Treatments alternating every third night were also administered to batches of wild-type or *csf1r* mutant larvae mosaic for *pnp4a*:nlsVenus-V2a-NTR that were later assessed for iridophore ablations.

### In situ hybridization

Characterization of mRNA transcript distributions in whole mount and transverse vibratome sections followed [64]. For comparing distributions of *csf1a* transcripts and iridophores, larvae were imaged prior to fixation, processed individually and then the corresponding regions re-imaged after color development.

### RT-PCR and quantitative RT-PCR

For quantitative RT-PCR, single skins were collected from ∼9.0 SSL *bnc2* or *bnc2*/+ larvae and placed in either Trizol Reagent (Invitrogen) or RNAlater (Ambion). RNA was isolated using either Trizol or RNaqueous Microkit (Ambion), followed by LiCl precipitation. cDNA was synthesized with either Superscript III First-Strand Synthesis (Invitrogen) or iScript cDNA Synthesis Kit (Bio-Rad). Quantitative RT-PCRs were performed and analyzed with a StepOnePlus System (Life Technologies) using a Custom Taqman Gene Expression Assay for *kitlga* (Life Technologies) and the following Taqman Gene Expression Assays (Life Technologies): *csf1a*, Dr03432536_m1; *csf1b*, Dr03110811_m1; *gapdh*, Dr03436842_m1.

For RT-PCR of isolated iridophores, 10–14 SSL larvae were euthanized and 3 skins placed in PBS. Tissue was briefly vortexed to remove scales, then centrifuged and washed again in PBS. Skins were incubated 10 min at 37°C in 0.25% trypsin-EDTA (Invitrogen). Trypsin was removed and tissue incubated 10 min at 37°C in trypsin-inhibitor (Sigma T6414) with 3 mg/ml collagenase, and 2 µl RNase-free DNase I (Thermo Scientific), followed by 3 h at 28 C in a Benchmark Multi-Therm Shaker set to 800 rpm. Cells were washed in PBS and filtered through a 40 µm cell strainer (BD Falcon). Cell mixtures were placed on a glass bottom dish and examined on a Zeiss Observer inverted compound microscope. Individual iridophores were picked using a pulled capillary and Narishige 1M 9B microinjector then expelled directly into resuspension buffer from the Superscript III Cells Direct cDNA Synthesis Kit (Invitrogen). cDNA was synthesized from approximately 50 cells per sample and RT-PCR performed with the following primers designed to span introns (forward, reverse): *actb1*, ACTGGGATGACATGGAGAAGAT, GTGTTGAAGGTCTCGAACATGA; *pnp4a*, GAAAAGTTTGGTCCACGATTTC, TACTCATTCCAACTGCATCCAC; *csf1a*, TACACCTTCACAGAGCGTCAGA, CTTCGTTGGACTGTCCTCAATC; *csf1b*, AACACCCCTGTTAACTGGACCT, GAGGCAGTAGGCAGTGAGAAGA.

### Imaging and quantitative analyses

For time-course imaging of interstripe development, fish from *bnc2*/+, *ltk*/+, or *ednrb1a*/+ backcrosses were reared individually and imaged daily on a Zeiss Observer inverted compound microscope or an Olympus SZX-12 stereomicroscope, using Zeiss Axiocam HR cameras and Axiovision software. Individuals from *bnc2*/+ backcrosses were genotyped retrospectively for the *bnc2^utr16e1^* lesion [33].

For transgenic rescue experiments of *bnc2* mutant melanophores and xanthophores, larvae were viewed and imaged as described above. For assessing melanophore numbers, all melanophores were counted ventral to the horizontal myoseptum in a region bounded by the anterior margin of the dorsal fin and the posterior margin of the anal fin. For assessing xanthophore numbers and localization, xanthophore were counted at three separate locations along the anterior to posterior axis (posterior swim bladder, anus, center of anal fin) within the interstripe region (as marked by iridophores).

To quantify melanophore dorsal–ventral location in *csf1r* mutants mosaic for *pnp4a*:nlsVenus-V2a-NTR and uninjected controls, we measured the distance of each melanophore from the dorsal and ventral margins of the myotomes, then divided dorsal length by total distance. Positions were determined for all melanophores between the anterior of the dorsal fin and posterior of the anal fin. Regions were considered ablated when they lacked most iridophores.

For presentation, images were color-balanced and in some cases adjusted for color saturation to assist in visualizing xanthophores.

All statistical analyses were performed using JMP 8.0.2 (SAS Institute, Cary, NC). For analyses of xanthophore numbers, counts were square-root transformed prior to analysis to correct for unequal variances across groups.

## Supporting Information

Figure S1 Expression of *csf1r* reporter by xanthophores during the larval-to-adult transformation. Interstripe xanthophores autofluoresce in the same channel as GFP and coexpress an mCherry *csf1r* reporter (arrowhead). Individual shown is *Tg(csf1r:Gal4.VP16)^i186^* injected with a plasmid containing 4xUAS-mCherry, resulting in mosaic mCherry expression.
(TIF)

Figure S2 Pigment cell distributions in *csf1r* mutants. (A) Despite the absence of well-differentiated xanthophores over the flank, *csf1r* mutants often exhibited a few lightly pigmented xanthophores at the level of the horizontal myoseptum and lateral line nerve (dashed blue line). (A′) Higher magnification view of boxed region in A. (B) In an individual in which iridophores have been ablated and partially recovered, residual xanthophores remained confined to the horizontal myoseptum and did not enter the region from which iridophores had been lost (likely owing to csf1r requirements for xanthophore migration [22], [54]). Shown here is the same region of the larva shown in main text Figure 8G (d8), with higher magnification view of boxed region and residual xanthophores in B′. (C) Image showing distributions of iridophores and melanophores along the ventral trunk of an unmanipulated *csf1r* mutant (10.4 SSL). Most melanophores are centered in regions lacking iridophores. (C′) Schematic showing distribution of iridophores (blue) and melanophores (black). Larvae in A and B were treated with epinephrine immediately before imaging (not all melanosomes have contracted in A). Scale bars: in (B) 60 µm for (A and B); in (C) 400 µm for (C,C′).
(TIF)
