# Supplementary figures and images for: Interactions with Iridophores and the Tissue Environment Required for Patterning Melanophores and Xanthophores during Zebrafish Adult Pigment Stripe Formation
Source: PLoS Genet. 2013 May 30;9(5):e1003561. doi: 10.1371/journal.pgen.1003561 (PMC3667786; doi:10.1371/journal.pgen.1003561)

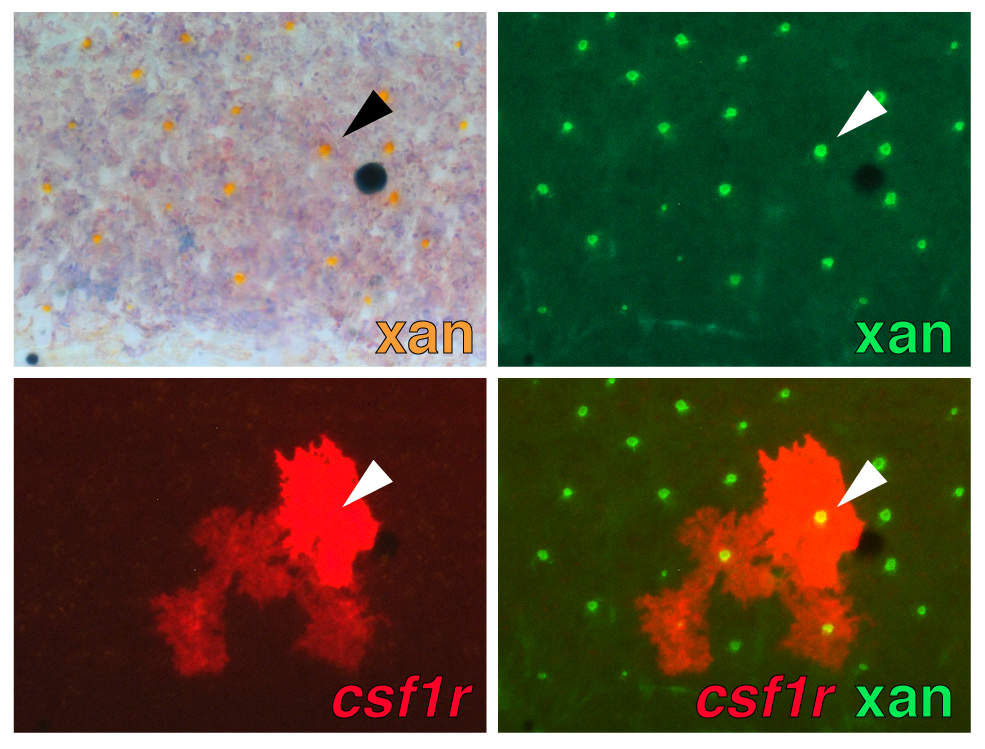

Supplement: Figure S1 — Expression of csf1r reporter by xanthophores during the larval-to-adult transformation. Interstripe xanthophores autofluoresce in the same channel as GFP and coexpress an mCherry csf1r reporter (arrowhead). Individual shown is Tg(csf1r:Gal4.VP16)i186 injected with a plasmid containing 4xUAS-mCherry, resulting in mosaic mCherry expression. (TIF) [file pgen.1003561.s001.tif]

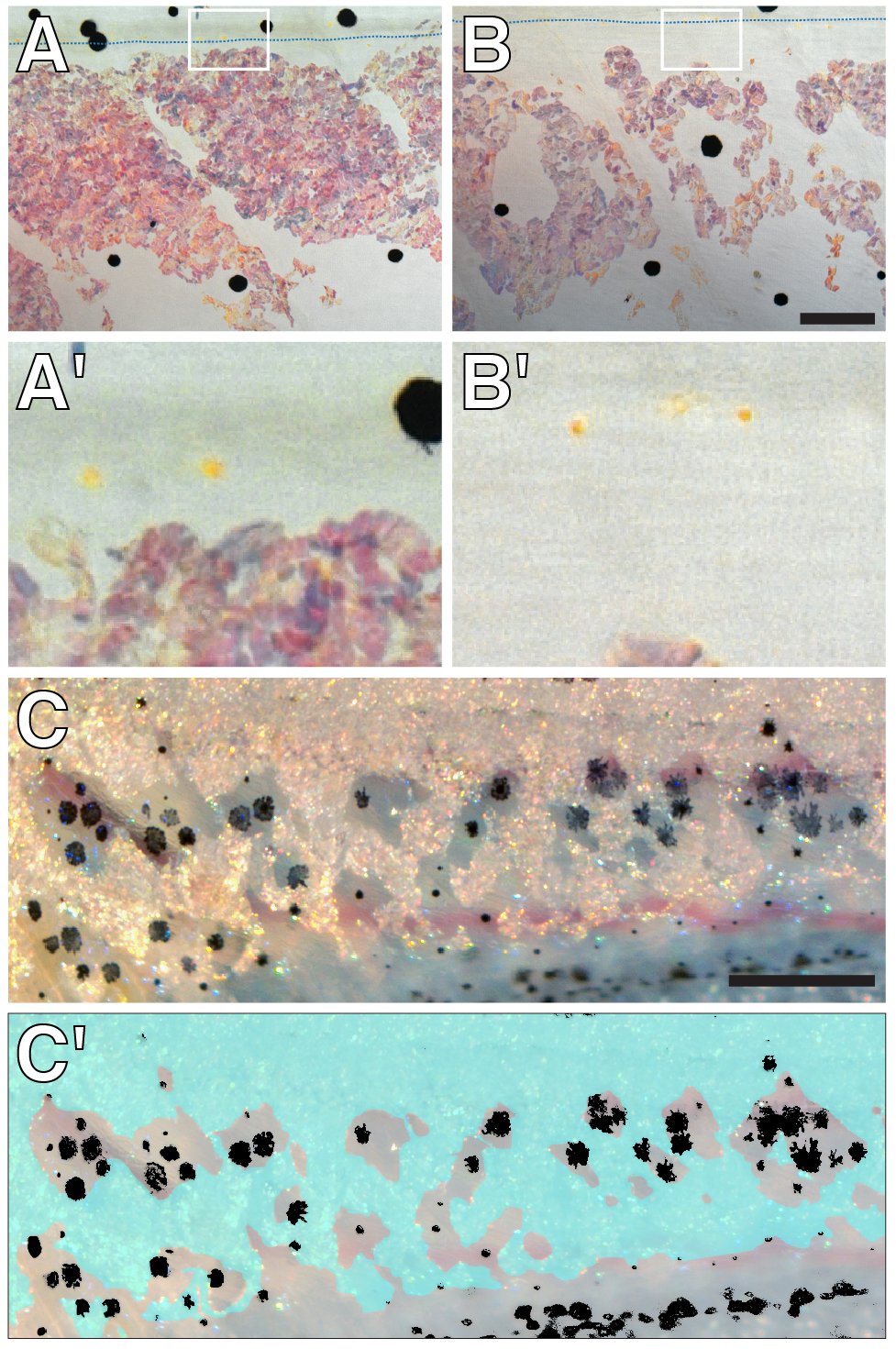

Supplement: Figure S2 — Pigment cell distributions in csf1r mutants. (A) Despite the absence of well-differentiated xanthophores over the flank, csf1r mutants often exhibited a few lightly pigmented xanthophores at the level of the horizontal myoseptum and lateral line nerve (dashed blue line). (A′) Higher magnification view of boxed region in A. (B) In an individual in which iridophores have been ablated and partially recovered, residual xanthophores remained confined to the horizontal myoseptum and did not enter the region from which iridophores had been lost (likely owing to csf1r requirements for xanthophore migration [22], [54]). Shown here is the same region of the larva shown in main text Figure 8G (d8), with higher magnification view of boxed region and residual xanthophores in B′. (C) Image showing distributions of iridophores and melanophores along the ventral trunk of an unmanipulated csf1r mutant (10.4 SSL). Most melanophores are centered in regions lacking iridophores. (C′) Schematic showing distribution of iridophores (blue) and melanophores (black). Larvae in A and B were treated with epinephrine immediately before imaging (not all melanosomes have contracted in A). Scale bars: in (B) 60 µm for (A and B); in (C) 400 µm for (C,C′). (TIF) [file pgen.1003561.s002.tif]
